# Sanitation predictors of childhood morbidities in Ethiopia: evidence from Dabat Health and Demographic Surveillance System

**DOI:** 10.1186/s12199-019-0801-0

**Published:** 2019-06-13

**Authors:** Zemichael Gizaw, Gashaw Andargie Biks, Mezgebu Yitayal, Geta Asrade Alemayehu, Kassahun Alemu, Tadesse Awoke, Adino Tesfahun Tsegaye, Amare Tariku, Terefe Derso, Solomon Mekonnen Abebe, Mulugeta Bayisa Chala

**Affiliations:** 1 Department of Environmental and Occupational Health and Safety, Institute of Public Health, College of Medicine and Health Sciences, University of Gondar, Gondar, Ethiopia; 2 Department of Health Service Management and Health Economics, Institute of Public Health, College of Medicine and Health Sciences, University of Gondar, Gondar, Ethiopia; 3 Department of Epidemiology and Biostatistics, Institute of Public Health, College of Medicine and Health Sciences, University of Gondar, Gondar, Ethiopia; 4 Department of Human Nutrition, Institute of Public Health, College of Medicine and Health Sciences, University of Gondar, Gondar, Ethiopia; 5 Dabat Research Center Health and Demographic Surveillance System, Institute of Public Health, College of Medicine and Health Sciences, University of Gondar, Gondar, Ethiopia; 6 Department of Physiotherapy, College of Medicine and Health Sciences, University of Gondar, Gondar, Ethiopia

**Keywords:** Housing condition, Waste management, Drinking water supply, Childhood morbidities, Dabat Health and Demographic Surveillance System

## Abstract

**Background:**

Failure to provide adequate sanitation services to all people is perhaps the greatest development failure. Globally, billions of people have no access to improved sanitation facilities. Though the link between sanitation and childhood morbidities is established globally, the evidence is limited in rural parts of Ethiopia. This survey was, therefore, designed to determine the prevalence of common childhood morbidities and to identify sanitation predictors in rural parts of northwest Ethiopia.

**Methods:**

A re-census reconciliation, which is a cross-sectional design, was employed from October to December 2014. All households found in the research and demographic sites were included as study subjects. A questionnaire and an observational checklist were used to collect data. Households’ sanitation performances, house type, illumination, household energy sources, water supply, and waste management were assessed. The occurrence of childhood morbidities was determined from the occurrence of one or more water, sanitation, and hygiene (WASH) preventable diseases. Multivariable binary logistic regression analysis was done to identify the association of sanitation factors with childhood morbidities on the basis of adjusted odds ratio (AOR) with 95% confidence interval (CI) and *p* value < 0.05.

**Results:**

About 575 (7.00%) of under-five children had hygiene- and sanitation-related diseases. Gastrointestinal and respiratory health problems accounted for 287 (49.91%) and 288 (50.09%), respectively. Childhood morbidities among under-five children were associated with poor housing condition [AOR = 1.27, 95% CI = (1.04, 1.54)], dirty cooking energy sources [AOR = 1.52, 95% CI = (1.22, 1.89)], volume of water below 20 l/p/d [AOR = 1.95, 95% CI = (1.19, 3.18)], and narrow-mouthed water storage containers [AOR = 0.73, 95% CI = (0.56, 0.96)].

**Conclusion:**

A significant proportion of under-five children had childhood morbidities in the study area. Housing condition, cooking energy sources, volume of water collected, and type of water storage containers were factors associated with the occurrence of childhood morbidities. Enabling the community to have the access to a safe and continuous supply of water and proper disposal of wastes, including excreta, is necessary with particular emphasis to the rural communities and semi-urban areas to reduce the occurrence of childhood morbidities.

## Background

Access to adequate water and sanitation is a fundamental need and a human right to all citizens. Increasing coverage of these essential services is vital for the dignity and health of all people and will significantly contribute to population welfare as well as the wealth and stability of countries. The failure to provide adequate sanitation services to all people is perhaps the greatest developmental failure [1].

Poor water, sanitation, and hygiene conditions cause a range of gastrointestinal and respiratory health problems, many of which are life-threatening. Each year, at least three million children under the age of five die due to environment-related diseases. Acute respiratory infections (ARIs) annually kill an estimated two million children under the age of five. As much as 60% of acute respiratory infections worldwide are related to environmental conditions [2]. Intestinal infectious diseases are also the most common cause of death among children [3]. Diarrhea and pneumonia are the most deadly gastrointestinal and respiratory health problems. In 2010, the global and regional estimate of the burden of diseases indicated that 7.6 million deaths among children younger than five years of age were caused due to sanitation-related infections. Among these deaths, 1.07 million (14.10%) and 0.75 million (9.90%) deaths were attributable to pneumonia and diarrheal causes, respectively. The greatest causes for these infections were water-, hygiene-, and sanitation-related [4].

Though the link between sanitation and childhood morbidities is established globally, the evidence is limited in rural parts of Ethiopia. This re-census was, therefore, designed to determine the burden of childhood morbidities and to identify sanitation predictors in rural parts of northwest Ethiopia, at Dabat Health and Demographic Surveillance System Sites.

## Methods

### Study design and settings

This cross-sectional study is a re-census employed for the purpose of reconciliation of the Dabat Health and Demographic Surveillance System (DHDSS). The surveillance system has been running since 1996 and has been collecting information on vital events (like birth, death, migration, and pregnancy registrations) and hygiene and sanitation conditions by updating every six months. The site is located in the Dabat district in the northwest part of Ethiopia. Dabat district was initially selected purposively as a surveillance site for its unique three climatic conditions, namely Dega (high land and cold), Woina Dega (midland and temperate), and Kolla (low land and hot). The choice was made with the assumption that there would be differences in morbidity and mortality in the different climatic areas. In 2014, the district had an estimated population size of 173,052 living in 26 rural and four semi-urban *kebeles* (the smallest administrative unit in Ethiopia). The DHDSS covers thirteen selected *kebeles* (four semi-urban and nine rural *kebeles*). A total of 67,385 people were living in the selected *kebeles* [5].

### Description of data collection process

Data about under-five children were extracted from DHDSS database which were collected by a re-census for the purpose of reconciliation. A re-census was employed in thirteen purposively selected *kebeles*. *Kebeles* were initially selected from three different agro-ecological zones for the purpose of showing differences in morbidity and mortality in the different climatic areas. Hence, we employed a re-census, and data collectors visited all the households in the selected *kebeles*. Data were collected using a pre-tested structured interviewer-administered questionnaire and an observational checklist. The living environment and housing conditions were observed, and photo cameras were used to take pictures by taking participants’ consent. Training was given for data collectors and field supervisors about the data collection tools, data collection procedures, and data collectors’ ethics. Mothers were interviewed about the most common childhood morbidities of their children and sanitation condition. Supervisors had also checked the completeness of all the filled questionnaires.

### Measurement of variables

Childhood morbidity, the primary outcome variable of this study, was defined as the occurrence of one or more gastrointestinal health problems (such as diarrhea, vomiting, and abdominal pain) and respiratory health problems (such as common cold, cough, pneumonia, asthma, and other sanitation-related respiratory problems). Childhood diarrheal disease was defined as having three or more loose or watery stools in 24 h [6]. The occurrence of the symptoms of the above morbidities in the past 2-week period was taken by asking the mothers/guardians. We used the local names of each disease to make clear for the mothers or guardians. Moreover, for the children who visited health institution, we checked the medication history to identify the specific types of diseases, and we asked the mothers or guardians to tell us the name of the diseases confirmed by the physicians. The sanitation predictor variables like housing condition, household energy sources, household light sources, domestic wastewater management practice, household’s solid waste management practice, household’s defecation practices, and drinking water supply system were defined and classified based on the minimum or indicators for access to basic sanitation set by the World Health Organization (WHO) and United Nations Children’s Fund (UNICEF) [7]. A housing condition was taken as good housing if the roof is covered by corrugated iron sheet (CIS); the floor is timber or concrete; rooms had two or more windows; the illumination system is adequate; and the energy was from clean sources like electricity. Illumination system was taken as good if the intensity of light is adequate and not emitting pollutants to the room like electricity and solar. Household cooking energy was taken as relatively clean energy if the source has no or minimal pollutants such as electric power, kerosene, and coal. Drinking water sources were taken as protected if the sources are protected from animal contact, flooding, and wind such as protected well, protected spring, protected rain catchment, and tap water. Households’ handwashing practice was taken as good if all family members washed hands after visiting the toilet, changing the baby’s diaper, or touching wastes and before eating and food preparation, otherwise taken as poor.

### Data management, processing, and analysis

Data were entered to Household Registration System (HRS2) version 2.1 and analyzed using Stata version 14.0. Univariable binary logistic regression analysis was used to choose variables for the multivariable binary logistic regression analysis, and variables which had a *p* value less than 0.2 by the univariable analysis were put into the final model. Variables which had significant association were identified on the basis of AOR with 95% CI and *p* value < 0.05. Pearson’s chi-square goodness of fit test was used to check model fitness.

Principal component analysis was used to determine the wealth index of rural households using asset data. In the final model, the common factor scores were summed and ranked into poorest quintile, second quintile, third quintile, fourth quintile, and richest quintile.

## Results

### Socio-demographic characteristics

A total of 8219 under-five children were included in this study. Of these children, 4149 (50.48%) were male. Four thousand eight hundred eighty-nine (59.48%) children were aged between 25 and 59 months. The overwhelming majority, 6491 (78.98%), of children were rural residents. Almost all, 8143 (99.08%), of the mothers interviewed were illiterate. One thousand seven (12.25%) of the households were the poorest by their economic status (Table 1).

**Table 1:** Demographic information of under-five children in DHDSS sites, Dabat, northwest Ethiopia, October to December 2014

Variables	Frequency	Percent
Sex of the child		
Male	4149	50.48
Female	4070	49.52
Age of the child in months		
0–6	752	9.15
7–12	895	10.89
13–24	1683	20.48
25–59	4889	59.48
Mothers’ or guardians’ education status		
Illiterate	8143	99.08
Primary school (grades 1–8)	44	0.54
Secondary school (grades 9–12) and above	32	0.39
Economic status of the household		
Poorest quintile	1007	12.25
Second quintile	1567	19.07
Third quintile	1893	23.03
Fourth quintile	1870	22.75
Richest quintile	1882	22.90
Residence of the child		
Semi-urban	1728	21.02
Rural	6491	78.98

### Housing condition

Six thousand one hundred seventy (75.07%) of the study participants lived in poor housing condition. Almost all, 8114 (98.72%), of the houses were earthen floor, and 2381 (28.97%) of the residential buildings were *tukul*, a house built with wood and grass. Five thousand eight hundred two (70.59%) of the residential buildings had no window(s). The general illumination system in 6323 (76.93%) houses was poor. Seven hundred forty-three (9.04%) of the households used open fire as a light source. The majority, 6897 (83.92%), of the households utilized dirty cooking energy, and fire wood was the commonest source (Table 2).

**Table 2:** Housing condition of study participants in DHDSS sites, Dabat, northwest Ethiopia, October to December 2014

Characteristics of housing	Frequency	Percent
Overall housing condition		
Good	2049	24.93
Not good	6170	75.07
Roof construction		
Wood and grass	2381	28.97
Corrugated iron sheet	5838	71.03
Floor type		
Earthen floor	8114	98.72
Concrete or cement floor	105	1.28
Availability of at least one window		
No	5802	70.59
Yes	2417	29.41
Illumination of the house		
Good	1896	23.07
Not good	6323	76.93
Light sources (multiple sources)		
Electricity	1896	23.07
Kerosene lamp	11	0.13
Sprite or Kuraz	61	0.74
Flashlight	6213	75.59
Open fire or wood	743	9.04
Candle	31	0.38
Solar	13	0.16
Cooking energy sources (multiple sources)		
Wood	6845	83.28
Animal dung	6700	81.52
Plant leaves/seed	1204	14.65
Charcoal	1267	15.42
Kerosene	8	0.10
Electricity	47	0.57
Condition of household’s cooking energy		
Dirty (biomass fuel)	6897	83.92
Relatively clean (charcoal, kerosene, and electricity)	1322	16.08

### Water supply

More than half, 4979 (60.58%), of the households fetched drinking water from unprotected sources. Unprotected spring was the commonest drinking water source in the study area, which accounted for 3409 (41.48%). Two thousand one hundred fifty-two (26.18%) of the households traveled more than 30-min round trip to fetch water, and almost all 8070 (98.19%) collected 20 l/p/d and below. Seven thousand seventy-one (86.03%) of the households used narrow-mouthed containers to store drinking water. Six hundred seventy-nine (8.26%) of the households treated drinking water at home. Sedimentation, 451(66.42%), was the commonest home-based water treatment method in the area (Table 3).

**Table 3:** Drinking water sources and home water handling among households in DHDSS sites, Dabat, northwest Ethiopia, October to December 2014

Variables	Frequency	Percent
Water sources (multiple sources)		
Tap water	1683	20.48
Protected spring	851	10.35
Protected well	750	9.13
Unprotected spring	3409	41.48
Unprotected well	95	1.16
River	1789	21.77
Condition of drinking water sources		
Unprotected	4979	60.58
Protected	3240	39.42
Time taken to collect water (round trip)		
< 30 min	6067	73.82
> 30 min	2152	26.18
Volume of water collected		
< 20 l/p/d	8070	98.19
> 20 l/p/d	149	1.81
Type of water storage container		
Narrow mouthed	7071	86.03
Wide mouthed	1148	13.97
Home-based water treatment		
Yes	679	8.26
No	7540	91.74
Home-based water treatment methods (multiple methods) (*n* = 679)		
Boiling	120	17.67
Chlorination	68	10.01
Cloth filtration	67	9.87
Sand or clay filtration	40	5.89
Sedimentation	451	66.42

*l/p/d* liter per person per day

### Waste management

In the study area, 2376 (28.91%) of the households used any type of latrine to defecate. Of the households who used latrine, 2140 (90.07%) households used traditional pit latrine. Seven thousand three hundred forty (89.31%) and 7030 (85.53%) respectively disposed domestic wastewater and solid wastes in open field. The handwashing practice of 6416 (78.06%) households was poor (Table 4).

**Table 4:** Waste management practices in DHDSS sites, Dabat, northwest Ethiopia, October to December 2014

Variables	Frequency	Percent
Household’s defecation practices		
Open defecation	5843	71.09
Any type of latrine	2376	28.91
Types of latrine commonly used (*n* = 2376)		
Traditional pit latrine	2140	90.07
VIP latrine	224	9.43
Septic tank	12	0.51
Household’s domestic wastewater handling practice		
Open field	7340	89.31
Sock pit	879	10.69
Household’s solid waste handling practice		
Open field	7030	85.53
Either burial or burning	1189	14.47
Household’s handwashing practice		
Good	1803	21.94
Poor	6416	78.06

*VIP* ventilated improved pit latrine

### Childhood morbidities

About 575 (7.00%) of under-five children had hygiene- and sanitation-related diseases. Gastrointestinal and respiratory health problems accounted for 287 (49.91%) and 288 (50.09%), respectively. Of the children who had hygiene- and sanitation-related diseases, 83 (14.43%) children had both gastrointestinal and respiratory health problems.

### Sanitation predictors of childhood morbidities

Housing condition, household cooking energy sources, household’s solid waste management practice, household’s domestic wastewater management practice, household’s defecation practices, drinking water sources, type of drinking water storage container, home-based water treatment, and volume of water daily collected were the variables fitted in the univariable binary logistic regression model. The variables having *p* value less than 20% were housing condition, household cooking energy sources, household’s defecation practices, type of drinking water storage container, and volume of water daily collected, which were selected for multivariable binary logistic regression analysis. By the multivariable binary logistic regression analysis, housing condition, household cooking energy sources, type of drinking water storage container, and volume of water daily collected were statistically associated with childhood morbidities (Table 5).

**Table 5:** Sanitation predictors of childhood morbidities among under-five children in DHDSS sites, Dabat, northwest Ethiopia, October to December 2014

Sanitation predictors	Childhood morbidities		COR with 95% CI	AOR with 95% CI
	Yes	No		
Housing condition				
Not good	412	5758	1.21 (1.0, 1.46)	1.27 (1.04, 1.54)*
Good	163	1886	1	
Household cooking energy source				
Dirty sources	449	6448	1.51 (1.23, 1.86)	1.52 (1.22, 1.89)***
Relatively clean sources	126	1196	1	
Human excreta management				
Open field	443	6085	1.16 (0.95, 1.42)	1.16 (0.93, 1.44)
Any type of latrine	132	1559	1	
Volume of water collected				
< 20 l/p/d	555	7515	2.10 (1.30, 3.39)	1.95 (1.19, 3.18)**
> 20 l/p/d	20	129		
Type of water storage container				
Narrow-mouthed container	506	6565	0.83 (0.641.08)	0.73 (0.56, 0.96)*
Wide-mouthed container	69	1079		

*AOR* adjusted odds ratio, *CI* confidence interval; *CIS* corrugated iron sheet, *COR* crude odds ratio, *l/p/d* liter per person per day

*Statistically significant at *p* < 0.05

**Statistically significant at *p* < 0.01

***Statistically significant at *p* < 0.001

This survey revealed that the occurrence of childhood morbidities was associated with housing condition. The odds of occurrence of morbidities was 1.27 times higher among children who lived in poor housing condition compared with children who lived in good housing condition [AOR = 1.27, 95% CI = (1.04, 1.54)].

This study revealed that household cooking energy sources were significantly associated with childhood morbidities. The occurrence of childhood morbidities was 1.52 times to be higher among children of households with dirty cooking energy sources compared with children of households with relatively clean energy sources [AOR = 1.52, 95% CI = (1.22, 1.89)].

As depicted by this survey, childhood morbidities were statistically associated with the volume of water daily collected. The odds of having childhood morbidities was 1.95 times higher among households who collected 20 l/p/d and below [AOR = 1.95, 95% CI = (1.19, 3.18). The current study had also discovered that the type of drinking water storage containers was associated with childhood morbidities. The odds of childhood morbidities was 27% less among children whose families stored drinking water in narrow-mouthed containers [AOR = 0.73, 95% CI = (0.56, 0.96)].

## Discussion

The 2-week period prevalence of childhood morbidities was 7.00 per hundred under-five children. The report of this re-census was lower than the reports of different community-based surveys like in Dabat, Ethiopia (33.70%) [8]; in Kenya (30.00%) [9]; in Wardha, India (59.90%) [10]; in Maharastra, India (34.70%) [11]; in Kolkata, India (52.45%) [12]; and in Tamil Nadu, India (71.00%) [13]. The lower prevalence of childhood morbidities in this study compared with the above findings might be due to seasonal variation.

The current survey found that the prevalence of gastrointestinal and respiratory health problems was 49.91% and 50.09%, respectively. Even though the prevalence for specific diseases was not exactly the same, other studies had also identified that diarrhea and respiratory problems were the major causes [8, 9]. A study done at demographic and health surveillance system in rural western Kenya reported that acute respiratory infections and diarrhea were the major causes of under-five morbidity [14].

This re-census had revealed that housing condition was associated with the occurrence of childhood morbidities. The result indicated that poor housing condition increases the likelihood of occurrence morbidities. This is due to poorly designed, constructed, and maintained buildings that create favorable condition for multiplication of disease-causing microorganisms and also contain toxic indoor air pollutants. Earthen floor and inadequate ventilation also increase interior moisture. Damp houses provide favorable conditions for microorganisms, molds, and vectors, which play roles in disease occurrence and transmission. Other similar studies also supported this fact [15–19].

This study showed that children of households with dirty cooking energy sources had more odds to develop morbidities. The association of dirty cooking energy usage and childhood morbidities can be due to the fact that dirty energy sources including wood, animal dung, or crop residues produce particulates, carbon monoxide, and other indoor pollutants, which can cause or aggravate ARIs, including upper respiratory infections such as colds and sore throats, and lower respiratory infections such as pneumonia. ARIs can also increase the risk of measles, malaria, and other diseases [20–22].

The occurrence of childhood morbidities was statistically associated with the volume of water daily collected. The prevalence of childhood morbidities was higher in households who collected water 20 l/p/d and below. This finding is in line with findings of other similar studies [23, 24]. This might be due to the fact that where the basic access service level has not been achieved, hygiene cannot be assured, which increases the occurrence and transmission of feco-oral diseases including respiratory health problems [23, 24].

The current study had discovered that the likelihood of occurrence of childhood morbidities was 27% less among children in households who stored water in narrow-mouthed containers. This can be justified that the safety of water stored in a narrow-mouthed container was higher than in wide-mouthed containers like buckets because contamination through dipping in smaller vessels like cups and jugs was minimized. Wide-mouthed containers are more prone to contamination because of their large open surface area. Other similar studies had also reported similar findings [25, 26].

## Limitation of the study

The occurrence of childhood morbidities was determined based on the report of mothers or head of the family. It was not confirmed by physicians. Due to this phenomenon, the current study might be affected by social desirability bias. The other limitation of this study was that separate analysis based on the nature and mechanisms of childhood morbidities (i.e., gastrointestinal health problems and respiratory health problems) was not done because of inadequate number of cases. Moreover, this study did not show the burden of childhood morbidities in different seasons of a year. In fact, socio-economic variables have effects on childhood morbidity, and we did not include them in the analysis. Hence, health data are limited in rural parts of Ethiopia, and with the limitations it has, this study will have a contribution to the policy makers and communities to design sanitation improvement and morbidity reduction strategies.

## Conclusions

A significant proportion of under-five children had poor water-, sanitation-, and hygiene-related morbidities in the study area. Housing condition, cooking energy sources, volume of water collected, and type of water storage container were identified as factors associated with the occurrence of childhood morbidities. There was a good start towards excreta management and water supply in the study areas; however, the quality of the facilities was not in a position of ensuring sanitation and disease prevention. Enabling the community to have access to safe and continuous supply of water and proper disposal of wastes including excreta is necessary with particular emphasis to the rural communities and semi-urban areas.

## Data Availability

Data will be made available upon request from the primary author.

## Abbreviations

AOR: Adjusted odds ratio
ARIS: Acute respiratory infections
CI: Confidence interval
CIS: Corrugated iron sheet
COR: Crude odds ratio
DHDSS: Dabat Health and Demographic Surveillance System
HRS2: Household Registration System
l/p/d: Liter per person per day
p: Proportion or prevalence
TB: Tuberculosis
UNICEF: United Nations Children’s Fund
VIP: Ventilated improved pit latrine
WASH: Water, sanitation, and hygiene
WHO: World Health Organization
